# An Accurate Calibration Means for the Phase Measuring Deflectometry System

**DOI:** 10.3390/s19245377

**Published:** 2019-12-05

**Authors:** Hao Han, Shiqian Wu, Zhan Song

**Affiliations:** 1 School of Machinery and Automation, Wuhan University of Science and Technology, Wuhan 430081, China; hao.han@siat.ac.cn; 2 Shenzhen Institutes of Advanced Technology, Chinese Academy of Sciences, Shenzhen 518055, China; 3 School of Information Science and Engineering, Wuhan University of Science and Technology, Wuhan 430081, China; shiqian.wu@wust.edu.cn; 4 Mechanical and Automation Engineering Department, The Chinese University of Hong Kong, Hong Kong SAR, China

**Keywords:** calibration, phase measuring deflectometry, 3D reconstruction, specular surface

## Abstract

Calibration is a critical step for the phase measuring deflectometry system. Existing calibration methods are mainly optimizing the calibration parameters with respect to the 2D re-projection error criterion. However, such a procedure cannot reduce metric errors in the practical application. Therefore, an accurate and practical calibration method is proposed. In which, conventional calibration means is first applied for the primary calibration. Then, a precise square planar mirror is used for the optimization of system calibration parameters. All the intrinsic and extrinsic parameters are considered as a global multi-objective optimization problem. Three metric error criteria are introduced to evaluate the 3D reconstruction accuracy of the reference mirror. Compared with classical calibration means, which apply the parameter optimization in 2D image space to minimize the re-projection errors, the proposed optimization approach is executed in 3D space directly. An experiment and comparison are conducted to verify that the proposed optimal calibration approach can effectively reduce the system deviation and to improve the system measurement accuracy.

## 1. Introduction

3D optical measurement has been widely used in various applications like industrial inspection, inverse engineering, robot navigation, and human–machine interaction, etc. [1,2,3,4,5]. Off-the-shelf commercial 3D scanners like structured light system [6,7,8] and laser 3D scanners [9] can perform well in the 3D measurement of diffuse surface or Lambertian surface. However, these 3D measurement techniques usually fail to deal with mirror-like specular surfaces subject to the strong reflectance. Instead of avoiding the specular reflection by coating the surface with powders, the phase measuring deflectometry (PMD) technique [10,11,12] takes full advantage of the surface reflective property, which captures the virtual image reflected by the target surface. By analyzing the deformed strip patterns captured by the camera, precise discrete slope variations of the test surface can be calculated. By integrating the slope data, 3D topography of the target surface can be reconstructed precisely [13,14,15].

In the PMD-based 3D measurement systems, the calculated surface slope and the integration step are highly sensitive to the system error, which is caused by the calibration accuracy [16,17]. Therefore, the calibration method of the PMD system is very important, which determines the system measurement precision directly. Mostly, the PMD system calibration performs by calibrating the camera and system geometric parameters respectively. The camera calibration is conducted to compute the term camera calibration parameters [18,19,20]. The system geometry calibration refers to determining the relation between the screen, the reference mirror, and the camera. In detail, this step aims to find correspondence between screen pixels, the mirror reflected points, and the camera pixels. Since the screen that used to display the strip patterns does not lie within the field view of the camera in the PMD systems, it is difficult to obtain geometrical relation between the camera and screen without auxiliary setup.

To solve the geometry calibration problem in PMD systems, existing approaches can be generally classified into four categories. (i) Using a laser tracker or a coordinate measuring machine to specify the positions of the camera, the screen, and the mirror [21,22,23]. Although the accuracy can be guaranteed by the usage of additional precise instruments, the operation is laborious and the equipment is very expensive. (ii) Using a reference planar mirror with markers printed on it [10,24,25]. These methods indirectly calibrate the pose between the screen and the camera by the position of the reference mirror. The mirror should be custom-made with precise markers. Besides, markers on the mirror may interrupt the reflected patterns. (iii) Using a marker-less mirror by changing its pose at least three different positions [26,27,28]. The pose of the screen can be estimated by optimizing all the pose parameters simultaneously with the application of bundle adjustment [27] process or orthogonality constraint [28]. (iv) By treating the system geometry calculation as an optimization problem [29]. Given initial values of the system geometry parameters, and the iteration procedure is introduced to reach an optimal value [30,31,32]. Such methods are usually sensitive to the initialization of system parameters and cannot guarantee global optimal results. Moreover, the optimization is based on the criterion of 2D image re-projection error, which cannot reflect final 3D reconstruction errors.

In this paper, a 3D criterion-based calibration method is proposed to improve the global calibration accuracy of the PMD system. The system is initially calibrated by traditional means with a flat mirror. Then, a planar mirror is used for parameter optimization. Based on the reference mirror, 3D metric error criteria are defined as the planarity error, the distance error, and the angular error. A multi-objective optimization problem is established by considering all the system parameters as variables. Using the primary calibration results as initial values, by minimizing the defined 3D measurement errors, the optimal calibration parameters can be computed finally. The results show, with a simple planar mirror and the proposed calibration method, system calibration accuracy can be greatly improved compared with conventional calibration methods.

## 2. Modeling and Initial Calibration of the PMD System

**Figure 1:**
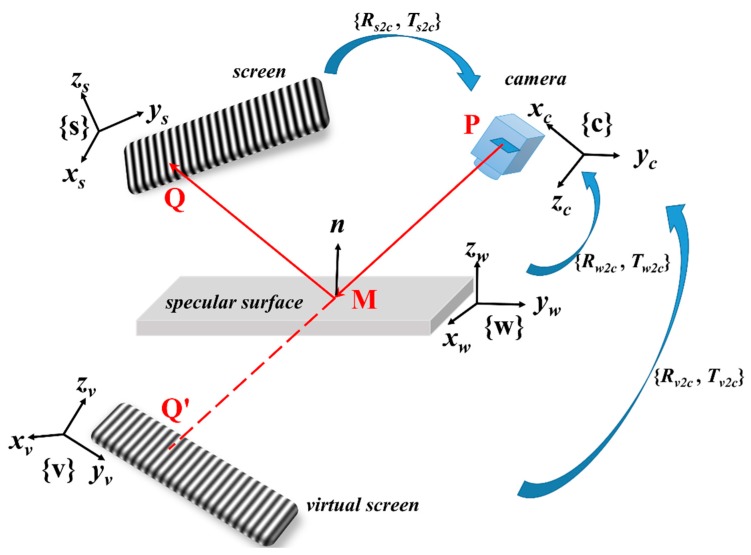
Principle of classical (Phase Measuring Deflectometry) PMD method to measure specular surface.

Figure 1 describes the basic principle of the PMD measurement system. The system is composed of a camera, a display screen, and the specular surface to be tested. Structured light patterns displayed by the screen are reflected by the targeted surface, and captured by the camera. The obtained images are the virtual images of the real screen. Then the surface x-slope and y-slope can be calculated by the system geometrical model and calibration parameters. Finally, the slope is integrated to obtain the 3D shape of the target surface. For simplicity, as shown in Table 1, the coordinate frames of the screen and the camera are defined as {s} and {c}, respectively. {v} refers to the virtual screen, which is the image of the real screen. The definitions of the concerned coordinate systems are listed in Table 1. In this paper, bold capital letters ***R*** and ***T*** are used to denote the rotation matrix and the translation vector. The subscript refers to coordinate transformation between the source and the target coordinate system, e.g., ***R_w_*_2*c*_** means the rotation matrix from world coordinate system {w} to the camera coordinate system {c}, and so on in a similar fashion.

**Figure 2:**
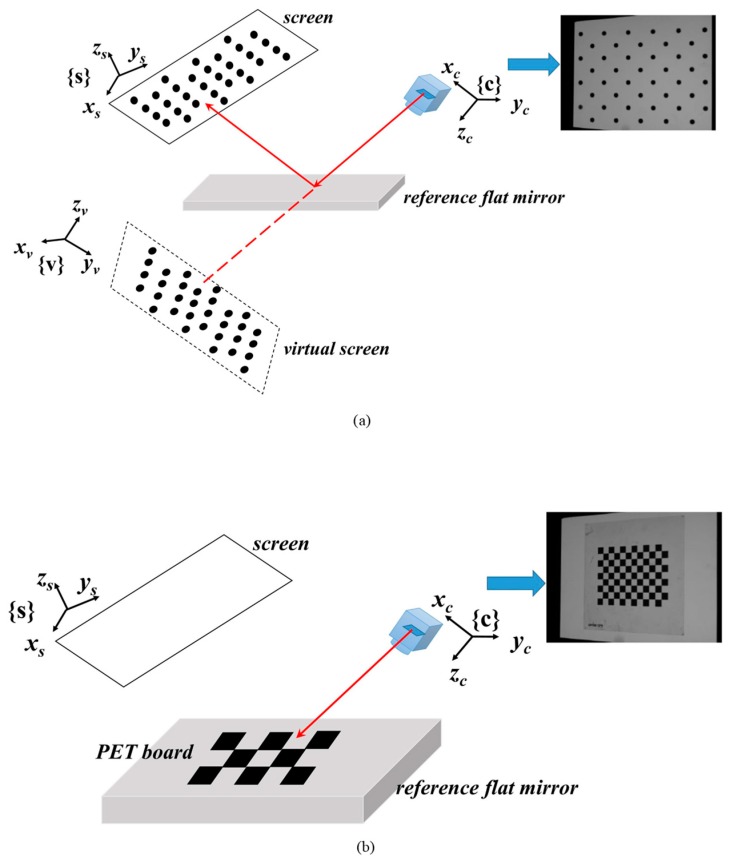
(**a**) Circular markers are displayed by the screen and used for the camera calibration. (**b**) The (Transparent Polyethylene Terephthalate) PET checkboard pattern used for the calibration of system geometrical parameters.

The fundamental principle of the PMD method is the law of reflection, which means the incident angle and reflected angle for any surface point should be exactly equal. For convenience, as shown in Figure 1, the camera can be assumed as an active observer. A ray that emitted from point *P* goes through the camera optical center, reflected by the specular surface at point *M*, and hits the screen at point *Q*. In the initial calibration procedure, the reference mirror is placed at more than three different positions. For each position, a pattern with asymmetric circular markers is displayed by the screen and captured by the camera to calculate the internal parameters as shown by Figure 2a. Because the virtual screen has further distance to the camera compared with the real target surface, the captured images are usually blurred subject to the defocus of the camera. The blurred calibration images make the localization of dot center inaccurate as well as the calibration results of camera. That’s also one reason to apply a global optimization for all the system parameters.

The virtual fiducials $Q^{\prime }$ is mapped to camera pixel *P*. Let ${Q^{\prime }}_{c}={\left[X_{c}\;Y_{c}\;Z_{c}\right]}^{T}$ denotes the coordinate in the frame {c}, and image pixel *P* is denoted as $p_{c}={\left[u_{c}\;v_{c}\right]}^{T}$ in image plane. According to the pinhole model, the normalized form of ***p_c_*** can be written as:

(1) $${\tilde{p}}_{c}=\left[\begin{array}{c} {\tilde{u}}_{c}\\ {\tilde{v}}_{c} \end{array}\right]=\left[\begin{array}{c} X_{c}/Z_{c}\\ Y_{c}/Z_{c} \end{array}\right].$$

Considering the lens distortions $D=[k_{1}\;k_{2}\;k_{3}\;p_{1}\;p_{2}]$, where $\left(k_{1},k_{2},k_{3}\right)$ refers to the radial coefficients and $\left(p_{1},p_{2}\right)$ refers to the tangential coefficients, the undistorted expression of ${\tilde{p}}_{c}$ can be expressed as:

(2) $$U({\tilde{p}}_{c})={\tilde{p}}_{c}\cdot (1+k_{1}r_{c}^{2}+k_{2}r_{c}^{4}+k_{3}r_{c}^{6})+\Delta ({\tilde{p}}_{c}),$$

where $r_{c}^{2}={\tilde{u}}_{c}^{2}+{\tilde{v}}_{c}^{2}$ and $\Delta ({\tilde{p}}_{c})$ refers to the tangential distortion vector that is expressed as:

(3) $$\Delta ({\tilde{p}}_{c})=\left[\begin{array}{l} 2p_{1}{\tilde{u}}_{c}{\tilde{v}}_{c}+p_{2}(r_{c}^{2}+2{\tilde{u}}_{c}^{2})\\ p_{1}(r_{c}^{2}+2{\tilde{v}}_{c}^{2})+2p_{2}{\tilde{u}}_{c}{\tilde{v}}_{c} \end{array}\right].$$

The homogeneous coordinate ${\bar{p}}_{c}$ of the undistorted point *P* can be expressed as:

(4) $${\bar{p}}_{c}=\left[\begin{array}{c} x_{c}\\ y_{c}\\ 1 \end{array}\right]=A\cdot U\left({\tilde{p}}_{c}\right),$$

where ***A*** is the camera intrinsic matrix, which can be expressed as:

(5) $$A=\left[\begin{array}{ccc} f_{u} & \alpha & u_{0}\\ 0 & f_{v} & v_{0}\\ 0 & 0 & 1 \end{array}\right].$$

For a complete model with lens distortions, there are 10 parameters to be estimated, i.e., $\left\{f_{u},f_{v},\alpha ,u_{0},v_{0},k_{1},k_{2},k_{3},p_{1},p_{2}\right\}$. The parameter of *α* refers to the skewness of the sensor axes, which can be assumed to be 0 for most modern imaging sensors. Intrinsic parameters of the camera can be estimated by a lot of existing calibration methods.

The extrinsic parameters between frame {v} and {c} are expressed as ***R_v_*_2c_** and ***T_v_*_2*c*_**, which can be mathematically represented as:

(6) $$s\left[\begin{array}{c} u_{c}\\ v_{c}\\ 1 \end{array}\right]=A\cdot \left[R_{v2c}|T_{v2c}\right]\left[\begin{array}{c} x_{v}\\ y_{v}\\ z_{v}\\ 1 \end{array}\right],$$

where *s* is a scale factor. Based on the maximum likelihood estimation algorithm [19], the internal and external parameters can be estimated by assessing the re-projection of the fiducials in frame {v} onto frame {c}, denoting the nonlinear mathematical function as ℜ, the minimization of the re-projection errors can be expressed as:

(7) $$\left[\begin{array}{lll} A & R_{v2c} & T_{v2c} \end{array}\right]=\arg\min\sum \left\|P-ℜ\left(Q^{\prime }\right)\right\|.$$

The structured light patterns captured by the camera are from the real screen frame {s}. Therefore, it is the real point *Q* that transformed into the camera frame {c}. To estimate the position and orientation of the real screen, a transparent Polyethylene Terephthalate (PET) calibration board is attached to the mirror at its last position and imaged by the camera, as shown in Figure 2b. Since the camera has been calibrated, the intrinsic parameters can be used to estimate the extrinsic parameters ***R_w_*_2*c*_** and ***T_w_*_2*c*_** by the following equation:

(8) $$\left[R_{w2c}\;T_{w2c}\right]=\arg\min\sum \left\|P-ℜ\left(M\right)\right\|.$$

According to the geometrical relation [33] and the Householder transformation [34], the relation between {s} and {c} can be calculated as:

(9) $$\{\begin{array}{l} \left(I_{3}-2n_{c}n_{c}^{T}\right)R_{s2c}=R_{v2c}\left(I_{3}-2e_{3}e_{3}{}^{T}\right)\\ \left(I_{3}-2n_{c}n_{c}^{T}\right)T_{s2c}=T_{v2c}+2d_{w2c}n_{c} \end{array},$$

where $I_{3}$ is a 3 × 3 identity matrix, and $e_{3}={\left[0\;0\;1\right]}^{T}$, $n_{c}$ is the normal vector of mirror respect to the camera frame {c}, $d_{w2c}$ refers to the distance between the calibration mirror and the camera optical center. They can be derived via ***R_w_*_2*c*_** and ***T_w_*_2*c*_** as:

(10) $$\left\{ \begin{array}{l} n_{c}=R_{w2c}(:,3)\\ d_{w2c}=\left|n_{c}^{T}\cdot T_{w2c}\right| \end{array}\right..$$

With the above operation and calculation, the initial camera and system geometrical parameters can be obtained. However, subject to the camera defocus, feature detection errors, manufacturing error of the transparent checkboard pattern and operating errors, etc., the initial calibration results of the PMD system are usually inaccurate. The following section will show how it can be optimized with a simple planar mirror.

## 3. Global Optimization of PMD System Parameters

**Figure 3:**
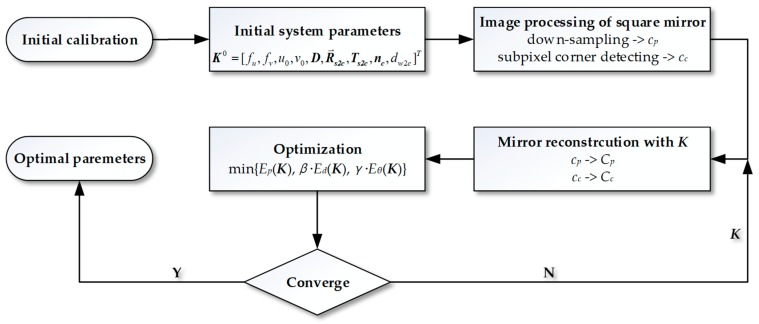
Workflow of the proposed optimal calibration method for PMD system.

Optimization of calibration parameters with respect to the 2D re-projection error criterion has been a standard step in existing calibration methods [16,26,29] for 3D optical measurement. However, the re-projection error cannot reflect real metric errors of the vision-based measurement system. More importantly, the 2D re-projection error based optimization procedure is only applied to the camera model parameters. But the factors of camera lens defocusing, structured light decoding error, and slope calculation errors, etc. are not considered. All these factors can make the final PMD calibration results inaccurate. That’s also the major motivation of this research, that a global optimization must be performed after the primary calibration so as to improve the system calibration accuracy. In this section, to improve the PMD system calibration accuracy, a practical 3D-based calibration procedure is introduced as follows. The underlying principle of the proposed method is to regard calibration of the PMD system as a global optimization problem. The primary calibration results are used as the initial values, and some objective functions are constructed to minimize the 3D metric deviation. By solving the nonlinear multi-objective optimization problem, the optimal parameters can be obtained. The workflow of the procedure is shown in Figure 3.

**Figure 4:**
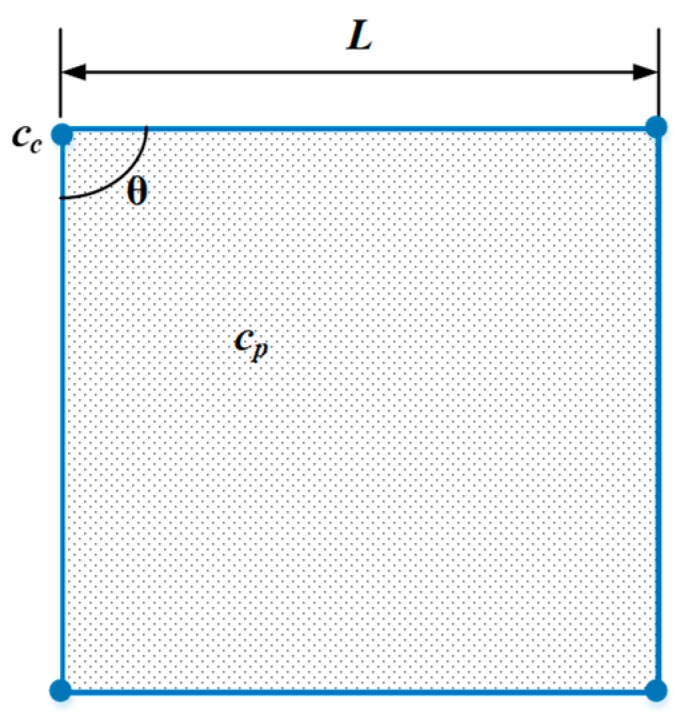
Illustration of the square mirror used for system parameters optimization.

The object used for calibration is rather simple as a square planar mirror, which is aluminum coated with λ/10 flatness to guarantee high planarity and precision. The first image displayed on the screen is a white pattern without any information, which is used to illuminate the mirror and make it visible for the camera. Based on this image, four corners (*c_c_*) of the mirror can be detected with subpixel accuracy, and the mask of the mirror area can be located in the image. To improve efficiency, a liner uniform down-sampling is applied and gets a set of image points (*c_p_*). Based on the reconstruction method and primary calibration parameters, 3D coordinates of *c_p_* and *c_c_* can be calculated and denoted as *C_p_* and *C_c_*, as shown in Figure 4.

Based on the reconstructed 3D information of the mirror, three metric errors are defined so as to construct the multi-objective optimization problem for accurate system parameter estimation. The adopted three objective functions are defined as follows.

### 3.1. Planarity Error

The square mirror used for reconstruction can be treated as a perfect plane. Regardless of the calibration derivations and reconstruction derivations, the planarity error of the *C_p_* should be zero. In general, a least-square fitting approach is applied to minimize the distance between the reconstructed points on the mirror surface. Given a plane, for each surface point, it has the same slope value in a certain direction. So we can use the slope values instead of planar fitting to describe the planarity term, which also makes the calculation more efficient. In details, the slope value can represent the planarity error by the following expression:

(11) $$E_{p}=\sum_{i=1}^{N}\left|s_{i}^{x}-{\bar{s}}^{x}\right|/N+\sum_{i=1}^{N}\left|s_{i}^{y}-{\bar{s}}^{y}\right|/N,$$

where *N* is the number of sampling points, $s_{i}^{x}$ and $s_{i}^{y}$ are slopes along the x and y-axis of the i-th sampling point, ${\bar{s}}^{x}$ and ${\bar{s}}^{y}$ are the corresponding mean value, respectively.

### 3.2. Distance Error

For each corner *c_c_*, its average distance to clockwise adjacent corners can be calculated and denoted as *d_j_*. There are four corners and the distance error is simply defined as:

(12) $$E_{d}=\sum_{j=1}^{4}\left|d_{j}-L\right|/4,$$

where, *L* is the nominal edge length of the mirror.

### 3.3. Angular Error

The planarity and distance error terms are still not enough to guarantee the reference plane will be well reconstructed. Considering the distortion caused by the affine transformation, the 3rd error term named angular error is defined. For each corner $C_{j}\in C_{c}$, by connecting it with the adjacent corners, all angles $\theta_{j}$ can be calculated, and the angular error can be defined as:

(13) $$E_{\theta }=\sum_{j=1}^{4}\left|\theta_{j}-\theta \right|/4,$$

where, the ground-truth value of θ is known to be 90 degree.

For the adopted PMD system, there are 19 parameters to be optimized totally, which can be expressed by a vector ***K*** as:

(14) $$K={\left[f_{u},f_{v},u_{0},v_{0},D,{\vec{R}}_{s2c},T_{s2c},n_{c},d_{w2c}\right]}^{T}.$$

Corresponding rotation vector is denoted as ${\vec{R}}_{s2c}$ by Rodrigues transformation to satisfy the orthogonality constraint, i.e., $R_{s2c}\cdot R_{s2c}^{T}=1$. The primary calibration results are used as the initial values of ***K*** and denote it as ***K***^0^. Therefore, a multi-objective optimization problem can be established as:

(15) $$\left\{ \begin{array}{l} \hat{K}=\arg\underset{K}{\min}\{E_{p}(K),\beta \cdot E_{d}(K),\gamma E_{\theta }(K)\}\\ s.t.\;lb\cdot K^{0}\leq K\leq ub\cdot K^{0} \end{array}\right.,$$

where the boundary coefficient *lb* and *ub* are empirically set to [0.9, 1.1], the weighting factors β and γ are used to balance the effects from three error criteria, which can be evaluated empirically to satisfy $E_{p}\approx \beta \cdot E_{d}\approx \gamma \cdot E_{a}$, e.g.,

(16) $$\left\{ \begin{array}{l} \beta =\frac{1}{L}\\ \gamma =\frac{1}{\theta } \end{array}\right..$$

To solve this multi-objective optimization problem, a lot of mathematical tools can be used. In this work, the “fgoalattain” function provided in the MATLAB optimization toolbox is used, which is based on the goal attainment method as described in [35]. Finally, the solved optimal system calibration parameters $\hat{K}$ are used for surface reconstruction. Since 3D metrics are used instead of 2D image re-projection errors, accurate measurement results can be promised as the experiments verified.

## 4. Experimental Results and Discussion

**Figure 5:**
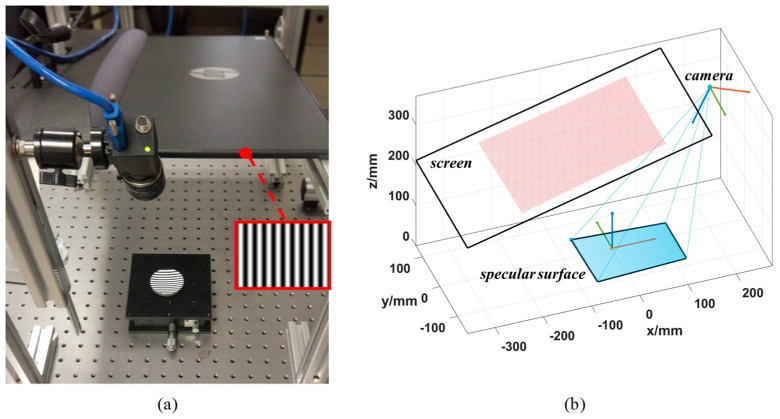
(**a**) The experimental setup of the PMD system; (**b**) Illustration of the PMD system model.

To verify the performance of the proposed calibration method, a PMD system is established as shown in Figure 5a. Dimension and relative positions of the setup is as shown by Figure 5b. The system consists of an LCD screen (HP 24 es 23.8-inch), which has a resolution of 1920 × 1080 and the pixel pitch is 0.2745 mm, an industrial camera (PointGray FL3-U3-32S2C-CS), which has a resolution of 2080 × 1552 pixel and the pixel size is 2.5 μm. The camera lens has a focal length of 12 mm. Distance from the object to the camera is about 360 mm. A sinusoidal phase-shifting pattern is adopted for the surface slope calculation as shown in Figure 5a. Virtual images of the displayed fringe patterns can be reflected by the mirror and then captured by the camera.

**Figure 6:**
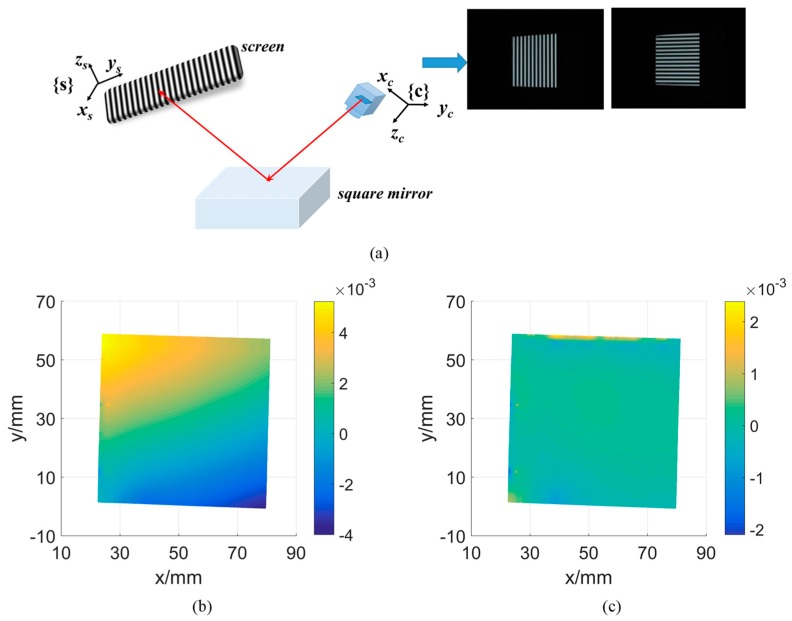
(**a**) The square mirror used for parameter optimization; (**b**) 3D reconstruction result with initial calibration parameters; (**c**) 3D reconstruction result with the optimal calibration parameters.

In the primary calibration step, the asymmetric dots pattern is displayed by the screen. By changing the posture of the reference flat mirror, 10 different views of patterns are acquired for camera calibration. While the last image was captured, without changing the position of the mirror, a transparent PET calibration board is attached on the mirror surface and captured by the camera. Following the method described in Section 2, the initial calibration parameters can be calculated. In the system parameter optimization step, a square mirror with a size of 60 × 60 mm is used as shown in Figure 6. Four corners of the mirror are detected from the image firstly. Then, with the primary calibration results, the mirror surface can be reconstructed to get its 3D model. Based on the reconstructed dense 3D points, a uniform down-sampling is applied to reduce the point number. Finally, three metric errors can be calculated. By solving Equation (15), the optimized system parameters can be calculated. Table 2 shows the parameters before and after optimization, namely ***K***^0^ and $\hat{K}$ respectively. The reference mirror is placed in the system as shown by Figure 6a. Measurement results of the reference mirror plane before and after optimization are shown in Figure 6b and Figure 6c respectively. And the three metric errors computed with two groups of calibration parameters are given by Table 3. We can see that the planarity by the optimized calibration parameters can be improved almost 10 times. The distance and angular errors also can be improved greatly after the optimization of system calibration parameters.

**Figure 7:**
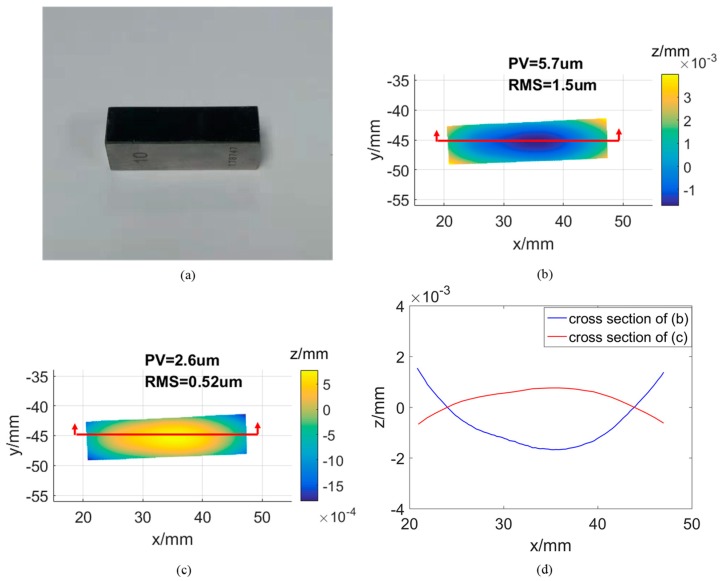
(**a**) A stainless gauge block; (**b**) Reconstructed surface by conventional method; (**c**) Reconstructed surface by proposed approach; (**d**) Cross section lines of the two 3D models.

In the first experiment, a stainless gauge block with a polished surface is used for evaluation, which has a size of 26 × 6 × 10 mm as shown in Figure 7a. The block surface is reconstructed twice based on the same structured light images with initial parameters and optimal parameters. So, the difference between the two measurement results can only be caused by the calibration error. 3D reconstruction results by the conventional and proposed method are as shown in Figure 7b and Figure 7c respectively. By fitting the reconstructed 3D points with an ideal plane, the peak and valley (PV) and root mean square (RMS) values are calculated as {5.7, 1.5} μm and {2.6, 0.52} μm respectively. To indicate the change of surface shape more clearly, a section line is selected and plotted as shown in Figure 7d, the blue and red lines are the profile of Figure 7b and Figure 7c, respectively. With the above comparison results, we can see that measurement results by the optimal system calibration parameters can be improved distinctly.

**Figure 8:**
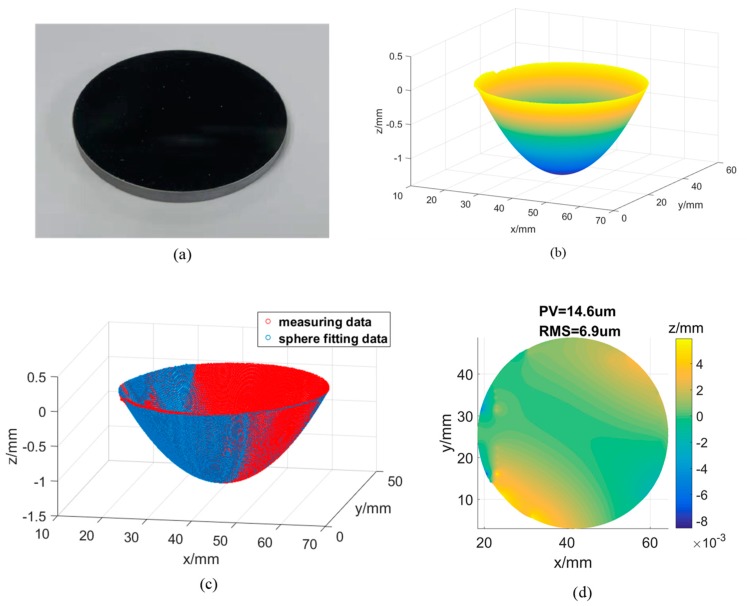
(**a**) A concave aluminized glass; (**b**) Reconstructed 3D model of the glass; (**c**) A sphere is fitted to the reconstructed surface points; (**d**) Distribution of the fitting error.

A concave aluminized glass is also used for the experiment as shown in Figure 8a. According to the specification provided by the manufacturer, its nominal aperture is 50 mm, and the radius of curvature of the concave mirror is 200 mm, with a tolerance of ±2%. Using the optimal calibration results, the reconstructed 3D model of the concave surface is as shown in Figure 8b. To visualize the 3D shape of the model, *z* axis is zoomed in. By fitting the reconstruction results with an ideal sphere as shown in Figure 8c, radius of reconstructed 3D model is about 199.12 mm, which locates within the metric tolerance. The PV and RMS error are calculated as 14.6 μm and 6.9 μm, respectively as shown in Figure 8d. In comparison, with the primary calibration results, the radius is calculated as 197.73 mm, the PV and RMS errors are 19.68 μm and 12.04 μm. Measurement accuracy improvement by the optimized system calibration results can be fully demonstrated.

**Figure 9:**
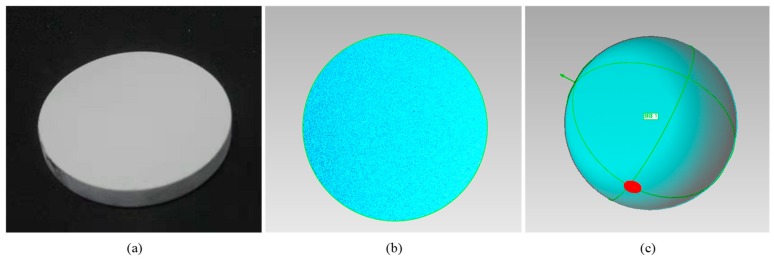
(**a**) The concave mirror after coating process; (**b**) Reconstructed 3D model of the concave surface by a structured light 3D scanner; (**c**) Sphere fitting of the concave surface.

To compare the 3D quality of the reconstructed shape with another 3D means, a commercial structured light 3D scanner (with nominal precision of 2.0 μ) was used to reconstruct the concave mirror and the result was shown in Figure 9. Subject to the mirror reflective surface, the structured light scanner cannot scan the mirror surface directly. To make the structured light scanner work, a coating operation was applied to the concave mirror surface to make it not reflective as shown in Figure 9a. The coating process is a usual procedure for the 3D scanners in industrial inspections, especially for the reflective or transparent surfaces. The reconstructed 3D model is as shown in Figure 9b. With a similar sphere fitting operation as shown by Figure 9c, the fitted radius is calculated as 206.9697 mm. In comparison, the result by the PMD system with optimized calibration parameters is much more close to the ground-truth data. In addition, compared with the 3D coordinate measuring method, the PMD method has the distinct advantage of non-contact and also much more efficient. Compared with structured light or laser 3D scanning techniques, the PMD method can work with a mirror-like surface directly. It has great potential in the online 3D inspection of mirror-like surfaces.

## 5. Conclusions

PMD is an important technique for the 3D measurement of mirror-like surfaces. Compared with conventional triangulation-based optical 3D measuring methods, the precision of the surface integration-based PMD methods is more dependent on the system calibration accuracy. In this paper, a simple and practical PMD system calibration approach is investigated to improve the system measurement accuracy. The system is calibrated by conventional means firstly. Then, a precise square planar mirror is used for the optimization of primary calibration parameters. Compared with traditional 2D image re-projection errors, three metric error criteria are introduced as the objective functions, i.e., planarity error, distance error, and the angular error. Using the primary calibration parameters as initial values, a nonlinear multi-objective optimization problem can be established and solved to obtain the optimal calibration parameters. Various mirror reflective surfaces are used for the experiments, and the results show that system measurement accuracy can be improved effectively. The approach is simple and effective, which is applicable not only for mono-PMD but also for stereo-PMD or multi-camera PMD configurations.

## Figures and Tables

**Table 1:** Notation of four coordinate systems.

Coordinate System	Notation
Camera coordinate system	c
Screen coordinate system	s
Virtual Screen coordinate system	v
World coordinate system	w

**Table 2:** Calibration parameters before and after optimization.

	*f_x_*/*f_y_*	*u*_0_/*v*_0_	*D*	${\vec{R}}_{s2c}$	$T_{s2c}$	*n_c_*	*d_w_*_2*c*_
			0.0457				
	4838.1586	1019.2370	0.1673	−0.0385	−398.5548	−0.4753	
***K*** ^0^	4837.3356	748.2347	−0.0023	0.8012	−145.6743	−0.0311	321.3857
			−0.0011	−0.0182	419.4638	−0.8793	
			5.3042				
			0.0683				
	4837.9968	1017.7158	0.3972	−0.0394	−397.5548	−0.4817	
K^	4837.1374	748.0554	–0.0005	0.7937	−146.7653	−0.0350	322.0015
			0.0014	−0.0179	418.1663	−0.8756	
			5.5530				

**Table 3:** Three metric errors computed with and without system optimization.

	*E_p_*	*E_d_* (mm)	*E_θ_* (°)
Initial parameters	0.0026	0.125	0.154
Optimal parameters	0.00028	0.026	0.031
